# The IRIDICA BAC BSI Assay: Rapid, Sensitive and Culture-Independent Identification of Bacteria and *Candida* in Blood

**DOI:** 10.1371/journal.pone.0158186

**Published:** 2016-07-06

**Authors:** David Metzgar, Mark W. Frinder, Richard E. Rothman, Stephen Peterson, Karen C. Carroll, Sean X. Zhang, Gideon D. Avornu, Megan A. Rounds, Heather E. Carolan, Donna M. Toleno, David Moore, Thomas A. Hall, Christian Massire, Gregory S. Richmond, Jose R. Gutierrez, Rangarajan Sampath, David J. Ecker, Lawrence B. Blyn

**Affiliations:** 1 Ibis Biosciences, an Abbott Company, Carlsbad, California, United States of America; 2 Department of Emergency Medicine, The Johns Hopkins University School of Medicine, Baltimore, Maryland, United States of America; 3 The Johns Hopkins Hospital Clinical Microbiology Laboratory, Baltimore, Maryland, United States of America; Wadsworth Center, New York Department of Health, UNITED STATES

## Abstract

Bloodstream infection (BSI) and sepsis are rising in incidence throughout the developed world. The spread of multi-drug resistant organisms presents increasing challenges to treatment. Surviving BSI is dependent on rapid and accurate identification of causal organisms, and timely application of appropriate antibiotics. Current culture-based methods used to detect and identify agents of BSI are often too slow to impact early therapy and may fail to detect relevant organisms in many positive cases. Existing methods for direct molecular detection of microbial DNA in blood are limited in either sensitivity (likely the result of small sample volumes) or in breadth of coverage, often because the PCR primers and probes used target only a few specific pathogens. There is a clear unmet need for a sensitive molecular assay capable of identifying the diverse bacteria and yeast associated with BSI directly from uncultured whole blood samples. We have developed a method of extracting DNA from larger volumes of whole blood (5 ml per sample), amplifying multiple widely conserved bacterial and fungal genes using a mismatch- and background-tolerant PCR chemistry, and identifying hundreds of diverse organisms from the amplified fragments on the basis of species-specific genetic signatures using electrospray ionization mass spectrometry (PCR/ESI-MS). We describe the analytical characteristics of the IRIDICA BAC BSI Assay and compare its pre-clinical performance to current standard-of-care methods in a collection of prospectively collected blood specimens from patients with symptoms of sepsis. The assay generated matching results in 80% of culture-positive cases (86% when common contaminants were excluded from the analysis), and twice the total number of positive detections. The described method is capable of providing organism identifications directly from uncultured blood in less than 8 hours. Disclaimer: The IRIDICA BAC BSI Assay is not available in the United States.

## Introduction

Bloodstream infection (BSI) and associated clinical sepsis represent a major source of mortality in the developed world, ranking as the 3^rd^ leading cause of death in Germany [1] and the 11^th^ in the United States [2]. With increasing numbers of elderly and immunocompromised patients as well as increased use of implanted medical devices, the incidence of sepsis is rising rapidly–by 75% between 1993 and 2003 in France [3]. With mortality rates ranging as high as 50% [3, 4], this situation constitutes a public health disaster. Average total costs associated with individual cases in Germany and the United States have been estimated at €23,297 and $22,100 respectively, with annual national burdens of €3.6–7.9 billion and $16.7 billion [4, 5].

The primary factor affecting the clinical outcome and financial burden of BSI is prompt treatment with appropriate antibiotics [1, 6, 7, 8, 9]. Mortality risk doubles with a 24 hour delay in provision of appropriate antibiotics in cases of bacteremia [10], and with a 12 hour delay in provision of antifungals in cases of candidemia [11].

Current culture-based methods used to detect and identify agents of bloodstream infection are inadequate. Incubation times of up to 5 days may be necessary to capture the majority of cultureable bacteria and fungi associated with BSI, though many infections can be detected after 24 to 48 hours [12, 13]. Further time is required to obtain identifications via MALDI-TOF, DNA sequence, or traditional biochemical analysis. These temporal delays leave practitioners with little choice but to treat all patients with suspected BSI empirically using broad-spectrum antibiotics. This strategy results in 15–30% of septic patients receiving inappropriate antibiotic therapies, which is, in turn, associated with a 2 to 5-fold higher mortality risk [6, 8, 14]. This risk derives from the empirical use of antibiotics that are ineffective for rarer organisms such as vancomycin-resistant enterococci (VRE) and *Candida* [14].

Standard culture-based methods are hampered by treatment with inhibitory antibiotics prior to sampling [7, 15] and are insensitive to non-cultivable or fastidious organisms such as *Coxiella burnetii*, *Tropheryma whipplei*, and species of genera such as *Bartonella*, *Rickettsia*, *Mycobacterium*, and *Nocardia* [16, 17]. Many of these organisms have been identified as causal agents of BSI by culture-independent molecular methods such as single-analyte PCR and 16S ribosomal gene sequencing; however, such methods lack either the coverage necessary to identify the diverse agents of BSI [9] or the sensitivity to consistently detect those agents directly from blood in the majority of cases [18, 19, 20].

Despite the low apparent sensitivity of existing broad-spectrum molecular methods with respect to culture—approximately 50% for many technologies [18, 19, 20]—such methods frequently yield additional positive results in culture-negative blood samples. These positive molecular detections are often confirmed in later cultures performed on samples taken from the same patients [1, 9, 12], are strongly correlated with sepsis-associated biomarkers [7, 21, 22], and have been shown to improve patient outcome when used to guide antibiotic therapy [1]. Molecular detection of bacteria in culture-negative specimens is correlated with antibiotic pretreatment [9, 23] and primarily identifies species known to be causal agents of BSI. These correlations all suggest that many culture-negative, PCR-positive detections represent culture insensitivity rather than a lack of specificity or clinical relevance on the part of molecular methods. This is supported by literature which widely reports that blood culture is positive in only 50% of cases where BSI is strongly suspected from a clinical standpoint [16, 24, 25].

There is a clear unmet need for rapid and sensitive molecular detection and identification of BSI agents directly from blood samples [2, 7, 20, 26, 27, 28, 29]. To meet this need, we have developed a universal lysis and DNA extraction method capable of sampling 5 ml of whole blood [24]. This is paired with conserved-site PCR primers capable of generating amplicons from >95% of the eubacteria and *Candida* species associated with human infection [30, 31] and PCR chemistry and cycling conditions compatible with high concentrations of background human DNA [24, 32, 33]. The method utilizes an automated desalting and DNA debulking platform to prepare amplicons for mass spectrometry [24, 34] and an electrospray ionization mass spectrometry (ESI-MS) platform capable of discriminating amplicon sequence variants from one or more different species present in a sample [35, 36, 37]. An onboard analysis computer is used to parse and report detections of 673 species of bacteria and *Candida* on the basis of multi-locus amplicon base composition signatures and quality controls including an external lysis control, internal PCR controls, external mass spectrometry standards, and signal strength and quality metrics [24, 31, 35].

In this study we explore the analytical sensitivity, specificity, robustness, reproducibility, and breadth of coverage of the IRIDICA BAC BSI Assay as performed on the IRIDICA System, and compare its performance to that of traditional culture-based methods in a collection of prospectively collected clinical whole blood specimens.

## Materials and Methods

### Microbiology

#### Culture and quantification of microbial stocks

Microbial stocks were obtained from ATCC (Manassas, VA) or clinical laboratories, grown on appropriate media and quantified by standard dilution and colony-count methods [31] at Ibis Biosciences (Carlsbad, California) or Zeptometrix (Buffalo, New York). Stocks were stored frozen in 15% glycerol at -70°C, thawed and diluted into test matrices, and again stored at -70°C or tested immediately.

#### Clinical sample collection and testing

Two hundred and eighty-five 5 ml whole blood samples were prospectively collected from consenting patients who presented to the Johns Hopkins Hospital Emergency Department and met at least two of the SIRS criteria for sepsis [38]. Samples were collected by phlebotomists in EDTA blood tubes following blood draws taken for standard-of-care culture analysis, using the same venipunctures. Samples were then blinded by replacement of identifying information with numerical identifiers by study coordinators, frozen, and transported to Ibis Biosciences on dry ice. Chart data and standard-of-care culture results were collected thereafter at Johns Hopkins Hospital by the study coordinators, associated with the respective blind sample identities, and then also blinded with respect to patient identity. Ibis Biosciences performed the IRIDICA BAC BSI Assay testing in the absence of chart and culture data. Paired specimens taken for clinical testing were processed per standard of care in the clinical microbiology laboratory at the Johns Hopkins Hospital, with no knowledge of the IRIDICA BAC BSI Assay results, and the results reported by the clinical microbiology laboratory were used here as the comparator. The two independently generated datasets (IRIDICA and culture) were brought together for comparison only after both methods had been performed and the data traceably documented.

#### Clinical laboratory culture and identification

The clinical microbiology laboratory used the Bactec FX (BD Diagnostics, Sparks, MD) continuous monitoring blood culture instrument. The bottles routinely used for clinical care included the Bactec Lytic/10 anaerobic bottle; the Bactec plus aerobic/F bottle containing resins for antibiotic neutralization (used for patients on antibiotics at the time of blood draw); and the Bactec Standard/10 aerobic bottle (an all-purpose medium that does not contain resins recommended for patients not on antibiotics at the time of blood draw). The laboratory blood culture procurement policy recommended that clinicians send a minimum of two sets of blood cultures with each set consisting of an aerobic bottle (either standard or aerobic plus) and an anaerobic lytic bottle, each inoculated with 10 mL of blood.

Aerobic gram-positive cocci and gram-negative rods were routinely identified using the Phoenix System (BD Diagnostics, Inc., Sparks, MD). Anaerobes were identified following recovery on routine media using either MALDI-ToF mass spectrometry (Bruker, Billerica, MA) or the RapID ANA II System (ThermoScientific, Waltham, MA). Gram-positive rods were identified using either the Phoenix System, MALDI-ToF MS, or cell wall fatty acid analysis using gas liquid chromatography, depending upon the genera isolated. *Candida* species were identified using recovery on Chromagar, morphology, and the Phoenix System yeast panels. Phenotypic resistance-typing methods were performed on isolates using the Phoenix System and the associated gram-negative (NMIC/ID-132) and gram-positive (PMIC/ID-105) panels (BD Diagnostics, Sparks, MD), extended-spectrum beta-lactamase E-tests (bioMerieux, Durham, NC), and the modified Hodge test. Molecular testing for *mecA*, *vanA* and *vanB* was performed using the Verigene BC-ID microarray system (Nanosphere, Northbrook, IL).

### Analytical testing

#### The IRIDICA System

PCR/ESI-MS testing was performed using the IRIDICA System components and their associated reagents (Abbott Molecular, Des Plaines, IL), including a high-volume bead-beating platform (IRIDICA BB), automated 5ml DNA extraction and PCR set-up platform (IRIDICA SP), PCR thermocycler (IRIDICA TC), automated amplicon desalting and DNA debulking platform (IRIDICA DS), automated electrospray ionization mass spectrometer (IRIDICA MS), and a control and analysis computer (IRIDICA AC). Functionality of these components has been previously described [24]. The system is capable of running 6 samples simultaneously in batches, and subsequent batches may be started 2.5 hours following initiation of the first. Most testing described here was performed by running multiple overlapping 6-sample batches on each testing day on each instrument. Each sample requires approximately 30 minutes of hands-on time for a single technician when run in batch mode (~2.5 hours hands-on time for a batch of 6 samples), involving reagent preparation, sample loading, transfer between instruments, and instrument decontamination. Hands-on requirements are interspersed temporally throughout the first 5 hours of the process and require a trained laboratory technician with competence in sterile technique, metered fluid transfer, vortexing, pipetting, use of a clinical centrifuge, and bar code scanning to track samples, equipment, users, reagents, and results. Time to first result averages approximately 7 hours.

#### The IRIDICA BAC BSI Assay

All testing utilized the IRIDICA BAC BSI Assay Kit, which includes a pre-filled PCR reaction strip encompassing 18 primer pairs in 16 wells. The primers target broadly conserved bacterial and *Candida* genes, 4 specific antibiotic resistance markers, *mecA*, *vanA*, *vanB* and *bla*_KPC_, and an extraction control target [31]. Reactions and cycling conditions were optimized to function in samples with a high human DNA background, and have been described previously [24]. Mass spectrometry signals from unfragmented amplicons were processed using analysis software and database elements designed to translate raw spectral data into base composition signatures for each detected amplicon. The software then performed signature matching between detected base compositions and multilocus species-specific signatures derived through analysis of type strains or surveys of whole-genome data from GenBank [35, 39].

#### Core test organisms

The IRIDICA BAC BSI Assay detects and identifies all target bacteria using a common set of primers and analysis algorithms. The same is true for all target *Candida*. On the basis of this unified functionality, a tiered approach to validation was followed [27, 31]. Four “core” organisms, which together utilize all primer pairs of the assay, were used for analytical studies designed to challenge the assay’s ability to detect target organisms at concentrations near the limit of detection (LOD) in various circumstances. The core organisms included *mecA*+ *Staphylococcus aureus* (MRSA), *vanA*+/*vanB*+ *Enterococcus faecium* (VRE), *bla*_KPC_+ *Klebsiella pneumoniae* (KPC), and *Candida albicans*.

#### Analytical studies

The limit of detection (LOD) of the assay was characterized for the 4 core organisms in 5 ml aliquots of both EDTA whole blood and sterile buffer (IRIDICA Negative Control, Abbott Molecular, Des Plaines, IL). Based on the resulting demonstration of comparable sensitivity in these 2 matrices, the LODs of 19 additional bacteria and *Candida* species were determined and confirmed in the sterile buffer. All LODs were measured by initial testing of 5 replicates at each of several 2-fold dilution steps, with the “determined LOD” being defined as the lowest concentration at which all 5 replicates were detected and correctly identified, including both the organism and any known drug resistance markers. This was followed by confirmation testing using 20 replicates at the determined LOD. The final (reported) LOD was defined as the lowest concentration at which at least 19 of 20 replicates were detected and correctly identified.

Another 47 species were tested at single concentrations in sterile buffer to confirm the designed breadth of coverage of the assay. Robustness was challenged through studies of potentially interfering substances (in EDTA whole blood), potentially cross-reacting organisms and carryover (in sterile buffer), and an in-house reproducibility analysis using multiple instruments, reagent lots and users (in EDTA whole blood, alongside samples prepared in sterile buffer, body fluids, and tissues). Carryover and cross-reactivity studies were performed at high titer while other studies were performed using the 4 core organisms at 3X LOD. Clinical sample performance was evaluated by comparing IRIDICA BAC BSI Assay results to culture results using 285 prospectively collected EDTA whole blood specimens from consented subjects with suspected BSI.

#### Assay controls

All analytical and clinical sample testing was carried out with the same control scheme using integrated and automated positive controls. These controls included both an extraction control target added to each sample and internal PCR calibrants formulated in each reaction well. PCR calibrants are synthetic competitive DNA constructs that contain the assay primer binding sites for one of the assay primer pairs contained in the well. The constructs are designed to produce unique base composition signatures which can be readily discriminated from the expected amplicons produced from target analytes. The calibrants are included in the PCR wells at defined concentrations and are used both to compete with low levels of background template and to gauge template input levels. Negative controls consisting of sterile buffer were run as a part of every set of 1–6 samples run concurrently on the platform. For positive detections in negative controls, any associated test results yielding the same detection were excluded. The rate of test validity was 96% over the course of the experiments described here. During analytical spiked sample studies, negative results paired with valid secondary detections of unspiked organisms (contaminants) were excluded on the basis of potential competitive interference.

### Comparison of IRIDICA BAC BSI Assay and culture results in clinical blood samples

As part of the standard of care, the collecting facility generally drew and tested two independent blood samples, each of which was inoculated into a blood culture bottle set consisting of one aerobic and one anaerobic bottle. Performance of multiple cultures is common practice to increase sensitivity and to discriminate between contamination events and clinically relevant detections. Contamination is generally characterized by single, unrepeated detections of common contaminants such as coagulase-negative staphylococci, viridans group streptococci, and species of the *Corynebacterium*, *Bacillus*, *Micrococcus*, and *Propionibacterium* genera. BSI is generally characterized by single or repeated detections of organisms strongly associated with BSI, such as MRSA, KPC, or VRE, or repeated detection of the same potential contaminant organism [40, 41, 42, 13, 43, 44].

In this study, culture results from a single blood culture bottle set were compared to IRIDICA BAC BSI Assay results from a single test sample, as only one sample was collected from each subject for testing on the IRIDICA System. In cases where the test sample was obtained from the same venipuncture as a sample used to inoculate a specific blood bottle set and that pairing was clearly documented, the associated culture result was used as the comparator. In cases where the specific link between test sample and clinical sample could not be confirmed (approximately one third of cases), the first reported culture result from the same day was used as the comparator. When available, results from secondary culture bottle sets and other species-specific pathogen identification results from patient chart data were used to assess IRIDICA BAC BSI Assay-positive, culture-negative discrepancies.

As discussed above, single positive detections of common contaminant organisms are of questionable clinical relevance, and the specificity of culture for these organisms is known to be low [40, 41, 42, 13, 43, 44]. Therefore, comparisons between culture and the IRIDICA BAC BSI Assay were made both with and without consideration of common contaminant organisms. When both culture and the IRIDICA BAC BSI Assay reported the presence of a bacterial species associated with one of the drug resistance markers targeted by the IRIDICA BAC BSI Assay, IRIDICA BAC BSI Assay resistance marker results were compared to phenotypic and/or genotypic resistance data reported by the clinical laboratory as part of post-culture standard-of-care isolate characterization. In cases where either the IRIDICA BAC BSI Assay or culture were negative for these bacteria, no resistance comparisons could be made because both methods report resistance markers or phenotypes only in association with specific bacterial detections.

## Results and Discussion

### Limits of detection and breadth of coverage

**Fig 1:**
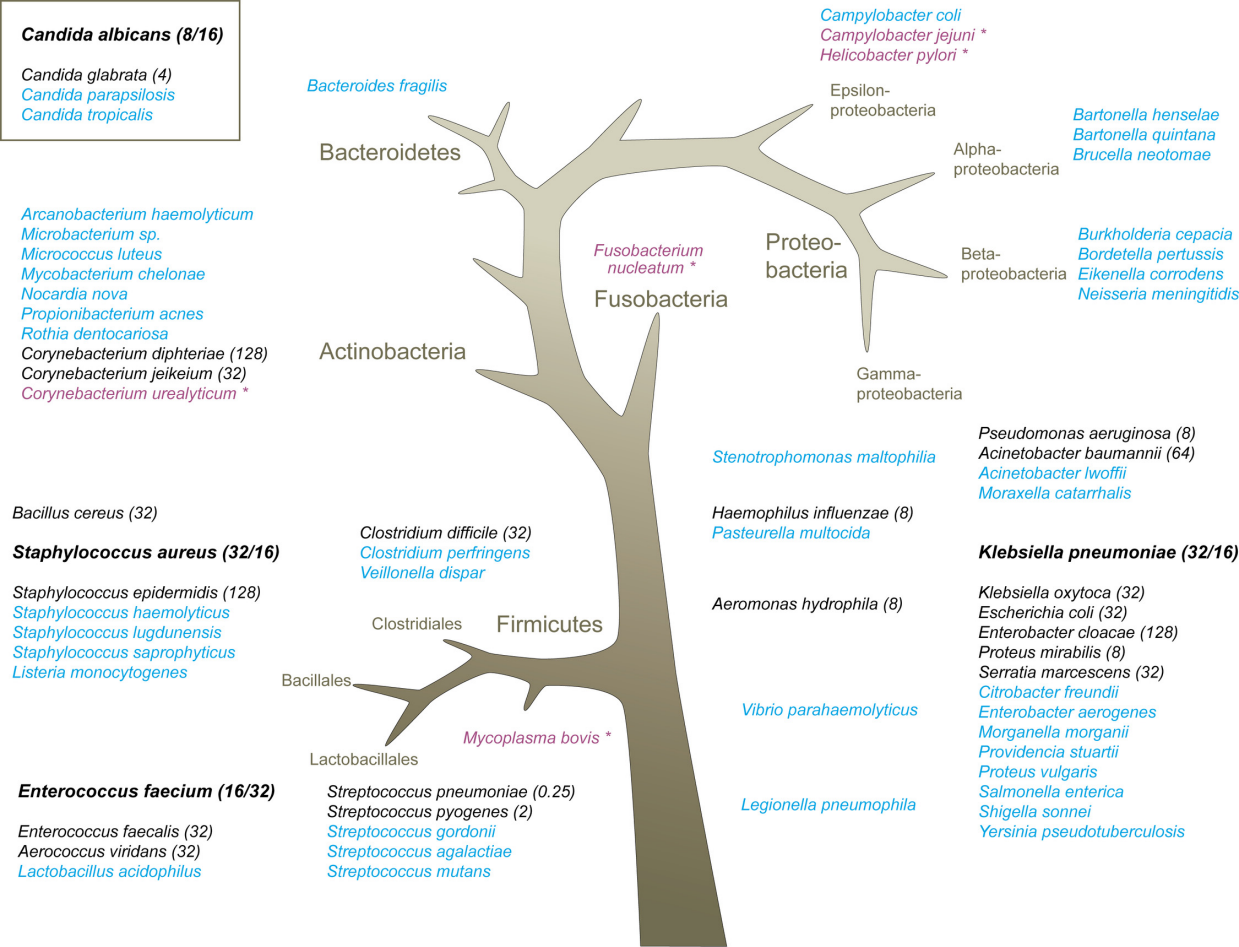
Organisms tested in analytical studies. The four core organisms are shown in bold, with LODs in blood and buffer shown in parentheses (blood LOD in CFU per ml / buffer LOD in CFU per ml). The LOD of 19 further organisms in buffer are also shown in parentheses. Remaining organisms shown in blue and purple were tested and detected in 2/2 replicates at either 100 CFU/ml (all except *Bacteroides fragilis*) or 200 CFU/ml (*Bacteroides fragilis*). *Indicates bioinformatic “worst-case scenario” organisms, for which the broad-spectrum primers used in the IRIDICA BAC BSI Assay are the least well-matched.

Limits of detection were measured for the four core organisms in both EDTA whole blood and sterile buffer matrices (see Fig 1). All four core organisms’ LODs were within 2-fold of each other between these two matrices, and further analytical studies were performed in sterile buffer except as noted.

The confirmed limits of detection of the IRIDICA BAC BSI Assay for 19 additional bacteria and *Candida* representing the breadth of coverage of the assay are shown in Fig 1. The geometric mean (log_2_) of the LODs for these species was 18.5 CFU/ml, with a range of 0.25–128 CFU/ml. This general limit of detection supports the theoretical ability of the assay to detect bacteria directly from the blood of most BSI patients, as the literature suggests that BSI patients carry approximately 1000 PCR-detectable genome copies of infecting organisms per ml of blood, in contrast to the very low levels of cultureable bacteria (1–10 CFU/ml) that can be grown directly from the blood of such patients [24]. The only species for which the assay LODs were < 4 CFU/ml were streptococci, which occur in chains and therefore have a high and variable genome:CFU ratio.

The assay successfully detected 47 additional diverse bacteria and *Candida* species in 2/2 replicates when tested at single concentrations. These organisms are shown in Fig 1, and include the species isolated by culture in >95% of BSI cases [30], organisms representing the full phylogenetic breadth of the assay [27, 31, 35], and five organisms identified as “bioinformatic worst-case” organisms (those to which the assay primers are most poorly matched) [27, 31].

### Robustness (cross-reactivity, interference, mixtures, and reproducibility)

Cross-reactivity was characterized with samples containing 10^5^ CFU/ml, copies/ml, or TCID_50_/ml of a variety of fungi, bacteria, and viruses which the assay was not designed to report. These included *Aspergillus flavus*, *Aspergillus niger*, *Clavispora lusitaniae*, *Candida kefyr*, *Caulobacter segnis*, *Pedobacter heparinus*, *Shewanella oneidensis*, *Streptomyces griseus*, influenza, parainfluenza, adenovirus, parvovirus, coronavirus, and herpesvirus. No cross-reactivity was observed.

The assay was able to detect and identify 3X LOD spikes of the four core organisms in EDTA whole blood supplemented with the potentially interfering substances listed in Table 1. For all but one substance, the test concentration corresponded to the recommendation in CLSI standards guidance [45]. The exception was bilirubin, for which all tested preparations were heavily contaminated with bacterial and fungal DNA. Despite the contamination, which prevented detection of one spiked analyte in one replicate through competitive interference at the original concentration (342 μmol/L), the assay still detected 11 of 12 spiked test organisms. All 12 spiked analytes were detected at 171 μmol/L bilirubin. The same contaminants were detected at both concentrations, indicating that extraction and PCR were fully functional and not directly affected by the bilirubin.

**Table 1:** Potentially interfering substances.

Substance	Test Concentration
Bilirubin	171 μmol/L
Hemoglobin	2 g/L
Triglycerides	37 mmol/L
White blood cells	15000 cells/μl
Glucose	1.2 mg/mL
Amikacin	136.8 μmol/L
Amphotericin B	3.3 μg/mL
Ceftazidime	117 μg/mL
Ciprofloxacin	30.2 μmol/L
Clindamycin	89.1 μmol/L
Doxycycline	67.5 μmol/L
Fluconazole	245 μmol/L
Gentamicin	21 μmol/L
Imipenem	249 μg/mL
Metronidazole	701 μmol/L
Piperacillin	1.24 mg/mL
Vancomycin	69 μmol/L
Dexamethasone	1.53 μmol/L
Dobutamine	15 mg/mL
Warfarin	32.5 μmol/L

Carryover testing was performed by testing adjacent negative and high positive samples. Positives were spiked with 10^7^ CFU/sample of either KPC, VRE, or both MRSA and *Candida albicans*. The test configuration provided 104 independent opportunities to observe carryover events between adjacent high titer and negative samples. No carryover events were observed.

Performance in samples containing multiple analytes was characterized by testing all 6 possible mixtures of two of the four core organisms at a 1:1 ratio at 3X LOD in triplicate. Because the IRIDICA BAC BSI Assay uses shared primers to detect related targets, competitive interference may prevent simultaneous detection of multiple targets. For this reason, this study was not intended to demonstrate that all analytes could be simultaneously detected and identified in mixtures, but rather that the presence of multiple organisms did not prevent the IRIDICA BAC BSI Assay from detecting and correctly identifying at least one of the components of a mixture. The assay successfully detected and accurately identified at least one of the two spiked analytes in 18/18 instances, and was able to detect and identify both spiked analytes in 15/18 instances. The three cases in which only one of the two spiked analytes were detected were all mixtures of MRSA and VRE–in two of these only MRSA was detected, and in one case only VRE was detected.

The within-laboratory reproducibility of the IRIDICA BAC BSI Assay bead-beating, sample prep, and PCR components (BB, SP, TC, Assay Strips, and related reagents) was characterized by testing panels of the four core organisms [27] at 3x LOD concentrations. Panels were tested by three operators using three lots of Assay Strips paired with three different IRIDICA BB, SP, and TC instruments over five days. The IRIDICA BAC assays [including the IRIDICA BAC BSI Assay (blood stream infection–as described in this paper), the IRIDICA BAC SFT Assay (sterile fluid and tissue), and the IRIDICA BAC LRT Assay (lower respiratory tract)] all share the same PCR strip configuration and formulation and may be run simultaneously on the IRIDICA System. The three lots of BAC Assay Strips tested in this protocol comprised one lot of each. Based upon the results of a previous DS/MS reproducibility study, the IRIDICA DS and MS instruments and reagents were not controlled as variables in this part of the study, though multiple DS and MS instruments and lots of reagents were used. All organisms were spiked into EDTA whole blood (BAC BSI Assay), saline bronchoalveolar lavage collection fluid (BAC LRT Assay), or the sterile buffer used to dilute normally sterile body fluid and tissue specimens (BAC SFT Assay) at 3x LOD. Either 500μl of bovine synovial fluid or 35 mg of porcine tissue was added to each IRIDICA BAC SFT Assay sample prior to bead beating. Reproducibility results are shown in Table 2. The average time from sample preparation to first result during the reproducibility study was approximately seven hours.

**Table 2:** Assay reproducibility at 3X LOD.

Variable (Identity)	# Detections/ # of Tests	Reproducibility
Operator 1	73/75	97.3%
Operator 2	72/75	96%
Operator 3	73/75	97.3%
Lot/Instrument 1	75/76	98.7%
Lot/Instrument 2	72/75	96%
Lot/Instrument 3	71/74	95.9%
Overall	218/225	96.9%

### Comparison of IRIDICA BAC BSI Assay and culture results in clinical blood samples

Two hundred and eighty-five clinical blood samples were analyzed by both culture and the IRIDICA BAC BSI Assay, generating 273 valid result sets. The validity rate of the IRIDICA BAC BSI Assay was thereby estimated at 95.7% in this clinical study. The IRIDICA BAC BSI Assay produced 85 organism detections, while culture produced 45. All organism detections are shown in Tables 3, 4 and 5, while antibiotic resistance marker/phenotype results are shown in Table 6.

**Table 3:** Comparison of IRIDICA BAC BSI Assay and standard-of-care culture results in clinical blood specimens from patients with suspected bloodstream infections, summarized by organism group (details in Table 4).

Organism Group	Matched Positive	BAC BSI Assay + / Culture –	BAC BSI Assay–/ Culture +	Matched Negative^B^
Gram-positive (including Mycoplasma)	15	11^A^^(5)^	2	207
Gram-negative	13	21^A^^(4)^	3	207
Unidentified bacteria	0	1	0	207
Yeast	2	1	0	207
Potential Contaminants (details in Table 4)	2	12^A^^(2)^	3	207
Other reportable organisms excluding potential contaminants (n = 550)	0	0	0	207

^A^These 11 culture-negative, IRIDICA BAC BSI Assay-positive detections were supported by later organism-specific ID data which identified the same species as agents of infection (as noted on the subjects’ charts). Numbers in parentheses indicate how many such cases were supported.

^B^Only samples which were negative for all analytes by both culture and the IRIDICA BAC BSI Assay were considered matched negatives (see discussion of additional detections in text).

**Table 4:** Comparison of positive IRIDICA BAC BSI Assay and standard-of-care culture results in clinical blood specimens from patients with suspected bloodstream infections, by organism. Possible identities of ambiguous IRIDICA BAC BSI Assay detections are separated by semicolons in cases where insufficient data was captured to provide species-level identification, and by slashes if the indicated organisms share identical reference signatures and cannot be discriminated. Commas separate multiple independent detections. These indicators are shown as they were reported by the IRIDICA BAC BSI Assay.

**Organisms, excluding potential contaminants**	**Matched Positive**	**BAC BSI Assay + / Culture –**	**BAC BSI Assay–/ Culture +**
*Azospirillum lipoferum*	0	1	0
Bacteria detected—No ID can be provided	0	1	0
*Bacteroides fragilis*	1	0	0
*Candida glabrata*	1	0	0
*Candida tropicalis*	1	1	0
*Citrobacter freundii; Klebsiella oxytoca*	0	1	0
*Ehrlichia chaffeensis*	0	1	0
*Enterobacter cloacae*	0	0	1
*Enterobacter cloacae* complex	0	2	0
*Enterococcus faecalis*	4	0	0
*Enterococcus faecium*	1	2^A^^(1)^	0
*Escherichia coli*	3	9^A^^(1)^	0
*Escherichia coli/Shigella flexneri/Shigella sonnei; Escherichia coli*	0	2	0
*Fusobacterium nucleatum*	0	1	0
*Klebsiella pneumoniae*	3	2^A^^(1)^	1
*Mycoplasma hominis*	0	1	0
*Providencia stuartii*	0	1	0
*Pseudomonas aeruginosa*	5	0	0
*Salmonella enterica*	1	0	0
*Salmonella* group B	0	0	1
*Shigella boydii; Escherichia coli*	0	1^A^	0
*Staphylococcus aureus*	9	5^A^^(3)^	1
*Stenotrophomonas maltophilia*	0	1^A^	0
*Streptococcus mitis/ pneumoniae; Streptococcus pneumoniae*	0	1^A^	0
*Streptococcus pneumoniae*	1	0	1
*Tsukamurella pulmonis*	0	1	0
Other reportable organisms excluding potential contaminants (n = 550)	0	0	0
Total Excluding Potential Contaminants	30	34	5
**Organism (potential contaminants only)**	**Matched Positive**	**BAC BSI Assay + / Culture –**	**BAC BSI Assay–/ Culture +**
*Propionibacterium acnes*	0	8	1
*Staphylococcus capitis*	1	0	0
*Staphylococcus epidermidis*	0	1^A^	0
*Staphylococcus saccharolyticus*	1	0	0
*Staphylococcus* species coagulase negative	0	0	2
*Streptococcus* species	0	1^A^	0
Viridans group *Streptococcus*	0	2	0
Other reportable organisms including potential contaminants (n = 652)	0	0	0
Total (including potential contaminants)	32	46	8

^A^These 11 culture-negative, IRIDICA BAC BSI Assay-positive detections were supported by later organism-specific ID data which identified the same species as agents of infection (as noted on the subjects’ charts).

Numbers in parentheses indicate how many such cases were supported. Two hundred and seven samples were negative for all analytes by both culture and IRIDICA (matched true negatives). These are shown in Table 3.

**Table 5:** Detailed results from samples with additional detections. Detailed data from all samples yielding unmatched positive results by either culture or IRIDICA BAC BSI Assay analysis in the presence of matched or unmatched detections by the other technology (additional detections). The matched positive results shown here in the second column are also represented in Tables 3 and 4, while the additional detections shown here in the rightmost two columns are not, because they have no valid comparator. See discussion of additional detections in text.

Sample #	Matched Positive Detections	Additional IRIDICA BAC BSI Detections	Additional Culture Detections
1	*Bacteroides fragilis*	NONE	*Streptococcus anginosus* group
2	*Candida glabrata*	NONE	Coagulase negative *Staphylococcus*, *Stenotrophomonas maltophilia*
3	*Enterococcus faecalis*	*Mycobacterium simiae*, *Propionibacterium acnes* (potential contaminant)	NONE
4	*Enterococcus faecium*	*Stenotrophomonas maltophilia*	NONE
5	*Klebsiella pneumoniae*	*Klebsiella variicola*	NONE
6	*Staphylococcus aureus*	*Klebsiella oxytoca*	*Streptococcus anginosus* group
7	NONE	*Arcanobacterium haemolyticum*, *Fusobacterium nucleatum*	*Finegoldia magna*

**Table 6:** Antibiotic resistance marker results summary.

Antibiotic Resistance Marker (Phenotype)	Matched Positive	BAC BSI Assay + / Culture –	BAC BSI Assay–/ Culture +	Matched Negative
*bla*_KPC_ (carbapenem resistance^A^)	0	0	0	0
*vanA/vanB* (vancomycin resistance^A^)	0	0	0	4
*mecA* (methicillin resistance^A^)	6	0	0	3

^A^Or equivalent–for example, oxacillin resistance for *mecA*.

Both culture and the IRIDICA BAC BSI Assay utilize single reagents to detect many or all targets. Therefore, the presence of detected organisms can mask the detection of other organisms through competitive interference. In culture, faster growing organisms can readily outcompete slower-growing organisms in culture bottles and obviate isolation of slower-growing organisms. In broad-spectrum PCR-based systems like IRIDICA, which use conserved-site primer pairs to amplify partially conserved regions from multiple species, targets with better matches to specific primer pairs may outcompete other species for amplification [27, 35]. This obscures meaningful interpretation of negative results in samples that are already positive for at least one analyte.

For the analysis presented here, results from both culture and the IRIDICA BAC BSI Assay were considered negative only if the sample yielded negative results for all analytes, while positive samples were considered positive for the reported analytes and indeterminate for all others. Positive detections which thereby had indeterminate comparators were assigned to the “additional detection” category in the final comparison (see Table 5).

Two hundred and seven samples yielded matched negative results for all analytes by both the IRIDICA BAC BSI Assay and culture methods. The IRIDICA BAC BSI Assay matched 32 of the 40 comparable detections reported by culture (80% positive agreement) and detected an additional 46 organisms in culture-negative samples. When potential contaminant organisms (listed at the bottom of Tables 3 and 4) were excluded from analysis, the IRIDICA BAC BSI Assay results matched 30 of 35 comparable detections reported by culture (86% positive agreement), and detected an additional 34 organisms not identified in culture. The majority of the IRIDICA BAC BSI Assay-positive, culture-negative detections identified either species commonly associated with BSI, including *Escherichia coli*, *Enterococcus faecium*, *Klebsiella pneumoniae*, and *Staphylococcus aureus;* or clinically relevant species unlikely to be detected by common culture bottle methods, including *Ehrlichia chaffeensis* [33] and *Mycoplasma hominis* [46]. Others were of fastidious species which occur as both pathogens and common environmental contaminants, such as *Mycobacterium simiae* [47]. Eleven of the 46 IRIDICA BAC BSI Assay-positive, culture-negative detections were supported by chart data showing positive detections of the same organisms and identifying them as causal pathogens in subsequent standard-of-care analyses of blood, biopsy, or respiratory specimens from the same patients. These are noted in Tables 3 and 4. The overall results obtained in this clinical sample study were consistent with those obtained in similar studies comparing the performance of the IRIDICA BAC BSI Assay with culture in separate populations of patients with suspected sepsis [48, 49].

Eight *Propionibacterium acnes* detections were made by the IRIDICA BAC BSI Assay and one by culture, none of which were matched. It is assumed that these represent contaminants introduced during venipuncture or sample preparation and reagent handling [40]. The IRIDICA BAC BSI Assay may be more sensitive to *P*. *acnes* contamination than culture methods. The organisms listed as potential contaminants in Table 4, along with other common blood culture contaminants, are notated as potential contaminants on IRIDICA BAC BSI Assay reports. It has been recommended that detection of these and other well-recognized common contaminant organisms in blood samples, whether through culture or molecular means, be confirmed with secondary samples rather than assuming clinical relevance [40, 41, 43]. During the clinical sample study, performed following the sterility and personal protective equipment recommendations of the manufacturer, 61 negative controls were tested and yielded no detections, supporting the assumption that detections in the clinical samples represented organismal DNA present in the sample.

In cases where both the clinical laboratory and the IRIDICA BAC BSI Assay detected an organism associated with one of the antibiotic resistance markers targeted by the IRIDICA BAC BSI Assay, standard-of-care resistance test results were compared with resistance marker detections reported by the IRIDICA BAC BSI Assay. In all 13 such cases, the standard-of-care results matched the resistance gene results from the IRIDICA BAC BSI Assay. Results are shown in Table 6.

## Conclusions

The data supports the ability of the IRIDICA BAC BSI Assay to identify diverse bacteria and *Candida* species directly from uncultured EDTA whole blood specimens, detecting 86% of the clinically relevant organisms isolated using traditional blood culture assays. The IRIDICA BAC BSI Assay is capable of providing such identifications within eight hours of sample collection, potentially allowing for the timely provision of appropriate targeted antibiotic therapy in cases of suspected bloodstream infection and sepsis.

The IRIDICA BAC BSI Assay cannot determine antibiotic resistance phenotypes, and is limited in terms of detecting resistance-associated genotypes to four common broad-spectrum antibiotic resistance elements–*mecA*, *vanA*, *vanB*, and KPC. Inclusive determination of resistance remains dependent on successful culture and subsequent phenotypic resistance typing of infecting organisms. For this reason, the IRIDICA BAC BSI Assay should be considered an additional tool in the diagnostic regime, not as a replacement for culture-based methods.

Similar to culture-based methods, the IRIDICA BAC BSI Assay is sensitive to both infecting pathogens and environmental microorganisms introduced during sample collection and preparation, necessitating rigorous phlebotomy technique, sterile laboratory handling, and use of negative controls. IRIDICA BAC BSI Assay results should be interpreted in the context of associated symptoms, risk factors, and other laboratory findings, in the same manner that current standard-of-care culture identifications are utilized.

The IRIDICA BAC BSI Assay yielded approximately twice as many positive detections as culture across the described set of 285 clinical blood specimens from patients with symptoms of sepsis. In the majority of cases, the IRIDICA BAC BSI Assay-positive, culture-negative detections were either of species commonly associated with BSI or of clinically relevant human pathogens that would be difficult to grow in blood culture bottles (*e*.*g*. *Ehrlichia chaffeensis*). Common contaminants (*e*.*g*. *Propionibacterium acnes*) were also observed in culture-negative specimens. Despite having access to only partial chart data, 11 of the 46 unmatched IRIDICA BAC BSI Assay detections were specifically supported by subsequent identifications and/or diagnostic data from independent specimens showing infection of the patient by the species initially detected by the IRIDICA BAC BSI Assay. Many of the IRIDICA BAC BSI Assay-positive, culture-negative detections thereby represent clinically relevant bloodstream infections which were missed by culture in the original paired specimen. This is consistent with literature suggesting that the sensitivity of culture is suboptimal [16, 24, 25]. The broad-spectrum nature of the IRIDICA BAC BSI Assay primers, paired with a signal analysis method capable of sensitive and specific detection and identification of one or more species signatures in samples with high background levels of human DNA, make it uniquely suited as a molecular test for bacterial and *Candida* DNA in blood samples.

### Ethics Statement

Clinical blood samples and associated clinical chart and microbiology data were collected under IRB protocol NA_00013251, “Evaluation of Universal Diagnostic Assays for Rapid and Accurate Pathogen Identification in Acute Care Settings”, approved by the Johns Hopkins Medical Institution internal review board, IRB-X. Verbal consent was obtained from all subjects or their designated surrogates, either in person or by telephone. Verbal consent was documented on copies of the consent script by the consenting physician or consent designee, and consent records were stored in a secure location. This procedure was approved by the JHMI IRB based on minimal risk of harm. Healthy subject blood used as matrix was provided by Biomed Supply LLC, Carlsbad, California and collected under FDA license.
